# The influence of group physical activity on oxytocin, empathy, affect, and stress in individuals with schizophrenia spectrum disorders in a single-arm, pilot and exploratory study

**DOI:** 10.1007/s44192-026-00420-9

**Published:** 2026-03-12

**Authors:** Marco Zierhut, Inge Hahne, Niklas Bergmann, Sofie Grässer, Max Alt, Laura Töbelmann, Josefa Wohlthan, Viktoria Huber, Thi Minh Tam Ta, Malek Bajbouj, Eric Hahn, Kerem Böge

**Affiliations:** 1 Department of Psychiatry and Neurosciences, Charité – Universitätsmedizin Berlin, Corporate Member of Freie Universität Berlin and Humboldt-Universität zu Berlin, Hindenburgdamm 30, 12203 Berlin, Germany; 2 Clinician Scientist Program, Berlin Institute of Health at Charité – Universitätsmedizin Berlin, BIH Academy, Berlin, Germany; 3 German Center of Mental Health (DZPG), Berlin, Germany

**Keywords:** Schizophrenia spectrum disorders, Physical activity, Oxytocin, Empathy, Affect, Stress

## Abstract

Research showed low plasma levels of the neuropeptide oxytocin in schizophrenia spectrum disorders (SSD), correlating with reduced empathy and negative symptoms. After oxytocin administration an increase in empathy was observed in healthy individuals, but the results are generally mixed. Endogenous oxytocin plasma levels increased after physical activity (PA). Due to the role of social factors in oxytocin’s effects, this study examined group-based PA in SSD and its effects. *N* = 34 individuals with SSD were recruited at Charité – Universitätsmedizin Berlin. Groups of up to six patients participated in a 30-minute manual-based PA session focusing on body tension and posture. Changes were assessed in plasma oxytocin, empathy, affect, and stress using self- and rater-based questionnaires. Oxytocin plasma levels significantly increased from baseline to post-participation in group-based PA. Moreover, significant reductions in negative affect (*Positive and Negative Affect Scale*), *symptom-related distress*, and increased contentment were found, as well as a trend toward a reduction of *Personal distress* on the *Interpersonal Reactivity Index* measuring empathy. In this exploratory study group PA resulted in elevations in endogenous oxytocin plasma levels. Secondary outcomes were mixed regarding empathy with only a trend toward reduction in *Personal distress.* Overall, group PA may have a stress-reducing effect, although it remains to be investigated whether oxytocin is the biological component in this process. Group PA could be a cost-effective adjunct treatment option for individuals with SSD. Future randomized controlled trials are needed to confirm effects and disentangle the interplay of PA, changes in oxytocin levels and symptoms in SSD.

**Supplementary Information:**

The online version contains supplementary material available at 10.1007/s44192-026-00420-9.

## Introduction

Schizophrenia spectrum disorders (SSD) affect approximately 24 million people globally [1]. Negative symptoms, among the core symptoms of schizophrenia spectrum disorders (SSD), are divided into five sub-domains: avolition, anhedonia, blunted affect, social withdrawal, and alogia [2–4]. They substantially impair social functioning and quality of life, are evident even before the first psychotic episode, and are associated with low remission rates and long-term morbidity [2]. Despite advances in pharmacotherapy, effective treatment options for negative symptoms remain limited [2, 5]. Recent models link negative symptoms to impairments in social cognition, particularly empathy, thereby contributing to the marked functional disability observed in SSD [6–9]. Cognitive empathy, a core deficit in schizophrenia, is closely related to negative symptoms and social functioning and involves medial prefrontal and temporal brain networks, suggesting shared behavioural and neurobiological substrates [10]. Consistent with this, lower plasma oxytocin levels in SSD are associated with both reduced empathy and more severe negative symptoms [11]. Accordingly, targeting social cognition is regarded as a key strategy for improving recovery in SSD [12].

Social cognition refers to the mental processes underlying social interaction [13]. Empathy, a central component of social cognition, encompasses both the cognitive ability to recognize others’ emotions (cognitive empathy) and the emotional capacity to resonate with them (emotional empathy) [14]. Individuals with SSD show marked impairments in empathy, particularly in empathic resonance, which substantially limits functional capacity and social participation [15].

Oxytocin, a neuropeptide involved in social behavior, social cognition, and stress regulation, has therefore received increasing attention in schizophrenia research [16]. In SSD, lower oxytocin levels are associated with greater negative symptom severity and reduced empathy [11]. ]. In healthy individuals, intranasal oxytocin enhances emotional and cognitive empathy via amygdala-modulated pathways [17]. On a neurobiological level oxytocin-induced improvements in social cognition are linked to increased connectivity within mesocorticolimbic dopamine circuits underlying social motivation and socioemotional processing [18]. However, clinical findings in SSD are mixed, with oxytocin effects depending on context, oxytocin levels, individual differences, and experimental paradigms [7]. This has prompted interest in approaches that increase endogenous oxytocin, which may offer a more naturalistic and potentially more effective way to influence social cognition. Given that empathy enhancement through exogenous oxytocin has been demonstrated in healthy individuals [19], it is important to examine whether these pathways can be engaged in SSD. Moreover, because patients are often sceptical about pharmacological treatments [19], non-pharmacological strategies that modulate endogenous oxytocin may be especially relevant.

Because oxytocin is closely linked to both empathy and stress regulation, stress-related neurobiological models are critical for understanding these impairments in SSD in exploratory approaches. People with SSD experience elevated stress levels, which are associated with negative symptoms such as blunted affect, avolition, and social withdrawal [20]. The extended stress-diathesis model integrates genetic vulnerability and developmental factors and highlights neuroendocrine dysregulation as a core mechanism [21–23]. Beyond cortisol [21, 24, 25], oxytocin has gained attention as a possible modulator of stress [26], capable of buffering stress responses [27], and enhancing social functioning [28], both of which are highly relevant to SSD. Consistent with this framework, mindfulness practice has been shown to increase oxytocin levels in both healthy individuals and people with SSD [29, 30]. In mindfulness-based group therapy (MBGT), oxytocin increased during the first session but decreased in plasma and saliva during the final session, with post-intervention plasma levels lower in the active group than in controls. This pattern suggests that oxytocin functions less as a direct marker of stress and more as a regulatory hormone shaped by social and contextual factors. Given oxytocin’s role in affiliative behavior and social cognition, these findings point to an interaction between stress regulation and social-affiliative neurobiology [31]. In a randomized placebo-controlled pilot study, participants with SSD receiving intranasal oxytocin before MBGT showed greater improvements in negative symptoms, psychological stress, and negative affect than those receiving placebo [8], further supporting oxytocin as a key mechanism linking stress, social cognition, and negative symptoms.

Several non-pharmacological interventions have been shown to modulate endogenous oxytocin. Mindfulness-based interventions increase circulating oxytocin in healthy individuals [29, 32] and MBGT in SSD produces dynamic oxytocin changes related to social novelty and stress regulation [30, 33, 34]. Yoga, which integrates mindful attention and embodied movement, increases oxytocin while improving emotion recognition and social functioning in SSD [35], reflecting combined interoceptive and social-affiliative mechanisms [36]. Beyond these approaches, physical activity (PA) also elevates endogenous oxytocin and improves symptoms and social functioning in SSD [37, 38], with aerobic exercise showing particular benefits for oxytocin and social-cognitive outcomes. Together, these findings identify mindfulness-, yoga-, and PA-based interventions as convergent behavioral pathways to enhance endogenous oxytocin.

Building on these findings, we want to further investigate the impact of alternative treatment options on endogenous oxytocin levels in SSD and its association with psychological stress parameters and thereby social cognition like empathy. This stress- and oxytocin-oriented perspective forms a conceptual bridge discussing oxytocin as a broader social-cognitive modulator and the rationale for targeting endogenous oxytocin through behavioral interventions.

From a phenomenological perspective, SSD can be conceptualized as a disorder of disembodiment affecting self-experience and intercorporality [35]. Intercorporality—understanding others through bodily resonance—suggests that PA may be particularly suited to improving empathy in SSD [35]. Group PA targeting posture and body tension may enhance bodily awareness, stimulate oxytocin release, and promote emotional resonance [39]. Acute aerobic exercise improves cognitive empathy in healthy individuals [40], and although most SSD studies have focused on individual PA [37], group-based PA appears especially promising for mental health outcomes in SSD [41].

While traditional aerobic exercise has demonstrated benefits for negative symptoms and cognition in SSD [37], high-intensity exercise may be poorly tolerated in SSD because of motivational deficits, negative symptoms, and comorbidities. Therefore, we developed a low-intensity, group-based PA intervention combining dynamic movements, mild cardiovascular activation, and social interaction. Group formats may be particularly beneficial in SSD, where low motivation and anticipatory pleasure deficits limit engagement in individual PA [42, 43], and where social withdrawal, isolation, and blunted affect may be ameliorated through oxytocin-mediated social processes [31].

Exercise-induced oxytocin may further modulate stress, fear extinction, social cognition, and recovery, depending on PA type and intensity, while also improving affect, body awareness, and well-being [39, 44]. Accordingly, we hypothesized that a 30-minute group PA session targeting body tension and posture would increase endogenous oxytocin levels in SSD.

Because oxytocin is a social hormone [16] and mindfulness- and yoga-based interventions are delivered in group settings [45, 46], we further assumed that a positively experienced group context would amplify oxytocin release and support empathy-related processes. In addition to examining oxytocin changes, we assessed stress, affect, empathy, and body awareness exploratively, as well as feasibility and acceptability of the intervention. Although the single-arm design and a potential Hawthorne effect limit causal inference, this exploratory approach provides a basis for subsequent controlled trials.

## Materials and methods

### Design and procedures

The study design is based on a single-center exploratory pilot trial with pre- and post-intervention measurements. Groups of up to six patients underwent a 30-minute supervised, manualized PA session focusing on body tension and posture. All outcomes were assessed before the session at baseline (T0) and directly after the session (T1) (see 2.4). Within-subject changes in oxytocin plasma levels constitute the primary outcome on an exploratory basis. Secondary outcomes included empathy, affect, stress, contentment, and body awareness, which also were analyzed exploratorily. Baseline negative symptoms were measured using validated self-rated and rater-based psychometric questionnaires to determine symptom severity for a better description of the patient population.

The authors assert that all procedures contributing to this work comply with the ethical standards of the relevant national and institutional committees on human experimentation and with the Helsinki Declaration of 1975, as revised in 2008. All procedures involving human subjects/patients were approved by the ethical committee of the Charité – Universitätsmedizin Berlin (EA4/196/19).

### Group physical activity

The intervention of this pilot study compromised a single group PA session aiming at positive changes through PA and group resonance, which was based on *Lederhosen Training*, a well-established German group sport training (Bavarian Sports UG, 2019). Developed a preceding iterative patient-involvingprocess with 12 SSD patients and a personal trainer, sessions were tailored to patient needs and abilities by feedback rounds, in order to create a positive, challenging and at the same time not overstraining intervention. In a previous study elevations in endogenous oxytocin levels were observed only in individuals who had no cortisol response to exercise, so moderate physiological activity had been suggested [47]. Therefore and based on literature [40], an optimal training duration of 30 min and the final selection of exercises was determined and manualized. The intervention primarily focused on body activation and mobilization and was held outdoors with an instructor and co-instructor to ensure inclusivity.

After an introductory round, the session started with three minutes of exercises to activate and stimulate blood circulation, followed by five minutes of mobilizing the spine and torso, focusing on muscle sensation. Afterwards, participants performed four minutes of balance exercises and ten minutes of exercises strengthening legs, arms, and upper back. The intervention concluded with three minutes of stretching and a verbal feedback round. During the exercises, the participants were positively reinforced by the instructor and reminded to focus on body sensations. A detailed description is available in the supplement.

### Participants

**Fig. 1:**
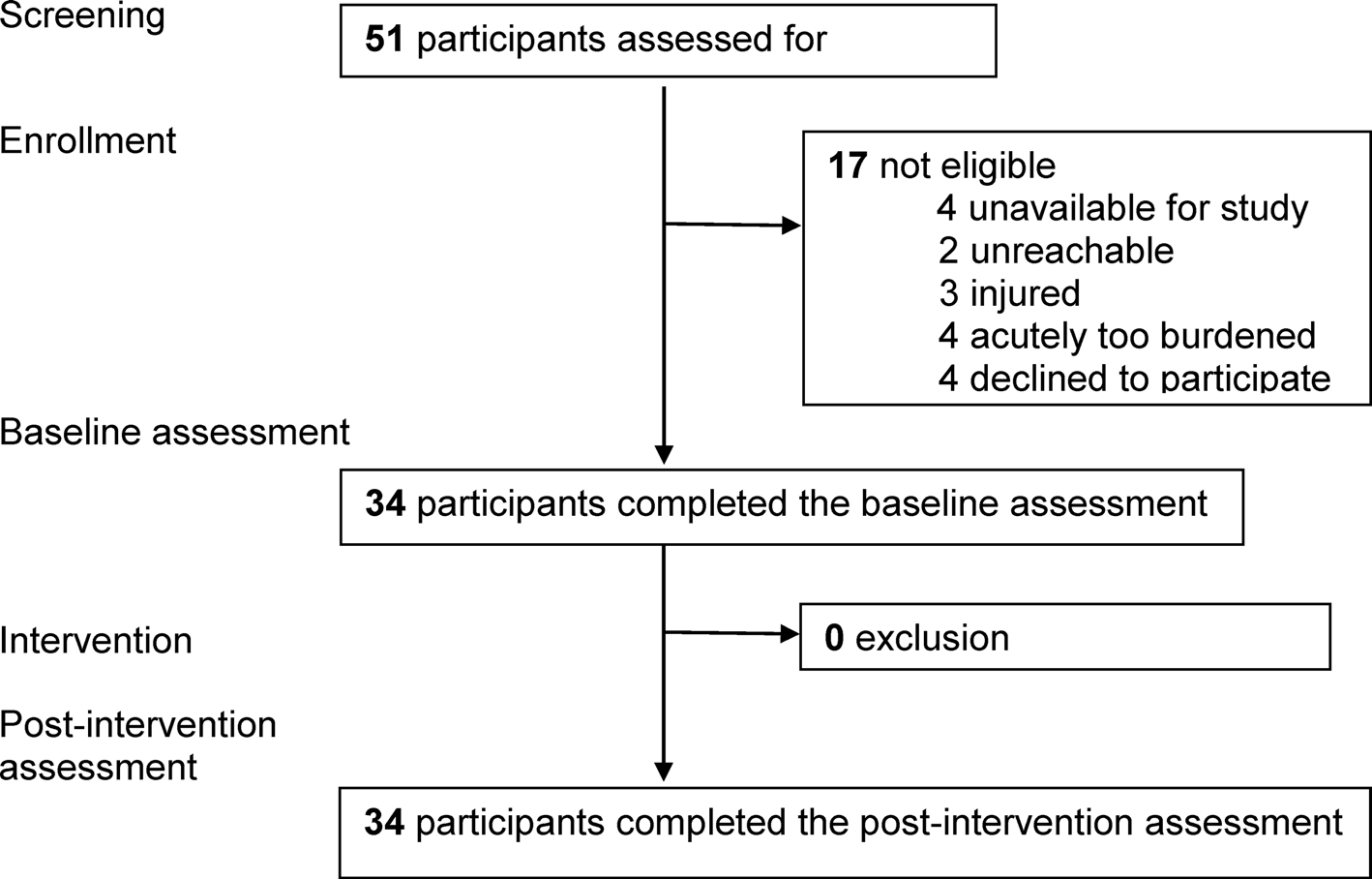
Study flow diagram of the recruitment process

Between July and October 2021, a multi-professional team recruited *N* = 34 individuals with SSD at the in- and out-patient clinic of the Department of Psychiatry and Neuroscience, Charité – Universitätsmedizin Berlin, Germany. Both men and women were included in mixed-gender groups. For inclusion, participants had to (a) be able to give written informed consent, (b) be aged between 18 and 65 years, (c) meet diagnostic criteria for SSD (ICD-10: F2X.X) assessed by a psychiatrist, (d) have sufficient German language proficiency, (e) and no recent (< 6 weeks) change in psychopharmacologic medication. Psychotropic medication and dosage were systematically recorded and remained stable during study participation. Negative pregnancy tests had to be demonstrated, as the associated hormonal changes could influence the measured oxytocin levels [48]. Exclusion criteria encompassed (a) a score > 6 on any item of the positive scale of the *Positive and Negative Syndrome Scale* (PANSS) [49], (b) acute suicidality assessed with the *Clinical Decision Support System* (CDSS) (item 8 > 1) [50], (c) current substance use other than nicotine or (d) the existence of severe physical impairments or neurological disorders. As our study involved only a single PA group session in which pre- and post-intervention effects were measured, the patients’ additional existing therapy, including ongoing psychotherapy and medication, was to remain unchanged, with only a few patients receiving psychotherapeutic treatment in addition to medication. Care was also taken to ensure that patients did not have psychotherapy sessions on the same day as the PA session. Sociodemographic and clinical data, including negative symptoms and social functioning measured by the *Social Performance Scale* (PSP), were assessed at baseline (T0) to describe the sample [51]. Written informed consent was obtained from all participants. The study flow diagram demonstrating the recruitment process is displayed in Fig. 1.

### Assessments

All study instruments at both time points are illustrated in Table 1.

**Table 1:** Overview: measurements across time points

	Rating type	T_0_	T_1_
Inclusion and exclusion criteria	Interview	X	
Sociodemographic data	Interview	X	
Positive and negative syndrome scale	Structured interview	X	
Self-evaluation of negative symptoms	Self-report	X	
International PA questionnaire - short form	Self-report	X	
Oxytocin plasma level	Biological parameter	X	X
Interpersonal reactivity index	Self-report	X	X
Multifaceted empathy test	Computer-based test	X	X
Positive and negative affect schedule	Self-report	X	X
Stress and contentment (visual analogue scale)	Self-report	X	X
Body mindfulness questionnaire	Self-report	X	X
Side effects and adverse events	Self-report		X

#### Negative symptoms

At baseline, positive and negative symptoms were rated using the two subscales (Positive Scale, Negative Scale) of the PANSS, a validated semi-structured and rater-based clinical interview. Each scale comprises statements on a seven-point Likert scale from 1 (absent) to 7 (extreme) to describe the individual’s symptom severity. The PANSS has good internal consistency, interrater reliability, and construct validity [49]. To account for subjectively experienced symptom severity, negative symptoms were additionally assessed by the *Self-Evaluation of Negative Symptoms* (SNS) at baseline with good internal consistency (Cronbach´s α of 0.87) [52]. It is a 20-item self-reported questionnaire with five subscales: *Alogia*, *Avolition*, *Anhedonia*, *Social withdrawal*, and *Diminished emotional range*. Participants answer on a Likert scale from 0 (strongly disagree) to 2 (strongly agree). The total score is the sum of the 20 items and ranges from 0 (no negative symptoms) to 40 (severe negative symptoms) [53].

#### Physical activity

The International Physical Activity Questionnaire Short Version (IPAQ-SF) [54] assesses the participants’ PA over the past week. It includes three items measuring time spent in PA in different intensities (mild, moderate, and vigorous) and the average daily sitting and walking time in minutes per day. We added two additional items on sleep quality and daily stress perception, rated on a 2-point Likert scale. The IPAQ shows good test-retest reliability of 0.80 [54].

#### Oxytocin plasma levels

Before and after the PA session, 10 ml venous blood samples were collected to assess oxytocin plasma levels. Blood samples were taken 30 min before and 30 min after PA, and stored on ice for a maximum of 20 min before being centrifuged and processed. The PA group always took place at 2 p.m. in the afternoon, as it was not possible to do so earlier because many SSD patients would not have been able to make it to the clinic in the morning. Patients were asked to fast and abstain from alcohol to rule out possible interactions with oxytocin levels. Ethylenediaminetetraacetic acid (EDTA) Monovette tubes (Sarstedt, Nümbrecht, Germany) containing aprotinin 400 IU/ml were used to avoid hormone degradation. Samples were centrifuged at 1300 g for 10 min at 4 °C. Supernatants were stored at − 20 °C until oxytocin levels (pg/ml) were analyzed by a highly sensitive (0.5 pg/ml range) and specific (< 0.7% cross-reactivity to a variety of peptides) radioimmunoassay with intra- and inter-assay variabilities of less than 10% (RIAgnosis, Munich, Germany) [55]. Prolactin, estrogen, progesterone, and lactate blood levels were measured to control for potential confounders with plasma oxytocin levels.

#### Empathy

The German version of the *Interpersonal Reactivity Index* (SPF-IRI), a widely used self-report measure for empathy [56], evaluates four dimensions (*Perspective taking*,* Empathic concern*,* Personal distress*,* Fantasy*) on a 1–5 Likert scale with high reliability, factorial validity, and item discriminatory power [56]. It is frequently employed in individuals with SSD [15, 57]. The *Multifaceted Empathy Test* (MET) assesses cognitive and emotional empathy using photographs of people in emotionally loaded situations to categorize by the test subjects in terms of emotions while recording response time, with high internal consistency, convergent, and divergent validity [58]. It has been utilized successfully in individuals with SSD [57]. Assessments were conducted using the software OpenSesame^®^ on a ThinkPad T14sGen1 with Windows 10.

#### Affect

Affect was measured using the *Positive and Negative Affect Schedule* (PANAS) [59], where participants rate applicability of adjectives to their current state on a scale from 1 to 5. The PANAS demonstrates strong internal consistency, ranging from 0.86 to 0.93 [60], and is widely used in individuals with SSD [44].

#### Stress, well-being and body awareness

Acute stress (*general stress* and *symptom-related distress*) was assessed by self-report visual analog scales, with ‘bubbles’ of increasing sizes representing different degrees [61]. Two additional questions on body well-being and contentment with oneself were included. The response format allows easy accessibility to individuals with cognitive impairments and is suitable for assessing within-session effects [61]. Additionally, the *Body Mindfulness Questionnaire* (BMQ) was used [62], consisting of two subscales (*Experiencing body awareness* and *Appreciating body awareness*). It is rated on a 6-point Likert scale and exhibits strong internal consistency, ranging from .91 to .93 [62].

#### Subjective side effects

After each session, side effects and adverse events were collected using questionnaires for cognitive, emotional, or somatic unpleasant experiences on a Likert scale from 0 (not applicable) to 3 (severe).

### Data management

Data collection and management were conducted pseudo-anonymously based on RedCap electronic case report files (eCRF) study software, entailing an authentication procedure with individual role management and secure encrypted connections [63].

### Statistical analysis

For this study, a power analysis and sample size calculation for the main hypothesis was performed with G-Power 3.1.3. Due to limited studies on changes in endogenous oxytocin levels from PA without reporting effect sizes [64], a conservative approach with a medium effect was adopted [38]. With a dependent samples *t*-test with *d* = 0.5, β = 0.80, and one-tailed *α* = 0.05, the sample size calculated was *N* = 34. Changes in oxytocin levels, empathy, affect, and body awareness were assessed exploratively using dependent samples t-tests with Cohen’s *d*_rm_ chosen as the effect size measure for within-session effects following recommendations from Lakens [65]. Stress and contentment were exploratively analyzed using Wilcoxon signed-rank tests due to non-normal distribution. For all outcomes, due to the study’s exploratory nature for these aspects, the false discovery rate was calculated to control for multiple testing [66]. In this study, we initially refrained from controlling for characteristics such as age and sex due to the small sample size in the context of a single-arm exploratory pilot study. Results should be interpreted cautiously, with trends warranting further investigation in more extensive randomized controlled trials. A significance level of α = 0.05 was set for all analyses. All p-values shown are values adjusted for false discovery rate. IBM SPSS Statistics 25 was used for data analysis.

## Results

### Sociodemographic and clinical data

A total of *N* = 34 participants were recruited, of which 29 were recruited from the outpatient- and five from the inpatient setting. The average age was 40.9 years, and the average duration of illness was 14.8 years. Of the participants, 82.4% were diagnosed with schizophrenia, 73.5% were male, 82.4% were single, and 88.3% had at least a secondary school degree or higher. With an average score of 15.9 on the PANSS *Negative Scale* and 15.4 on the SNS, the average severity of negative symptoms of the cohort can be categorized as mildly ill [53]. The PA level was low to moderate in 94.1% of the participants. Sociodemographic and clinical data can be found in Table 2.

**Table 2:** Sociodemographic and clinical data

Variables	Summary statistics^a^
Age (years) ^a^	40.9 (13.1)
Diagnosis	
Schizophrenia (F20)^b^	28 (82.4%)
Schizotypal disorder (F21)^b^	1 (2.9%)
Brief psychotic disorder (F23)^b^	2 (5.9%)
Schizoaffective disorder (F25)^b^	3 (8.8%)
Duration of illness (years) ^a^	14.8 (11.3)
Sex	
Male^b^	25 (73.5%)
Female^b^	9 (26.5%)
Family status	
Single^b^	28 (82.4%)
Married^b^	4 (11.8%)
Divorced/widowed^b^	2 (5.9%)
With children^b^	9 (26.5%)
Years in school ^a^	11.6 (1.4)
Highest education level	
Primary school^b^	4 (11.8%)
Secondary school^b^	11 (32.4%)
A-level^b^	14 (41.2%)
Apprenticeship^b^	1 (2.9%)
Studied^b^	4 (11.8%)
Occupation	
Employed^b^	9 (26.5%)
Self-employed^b^	5 (14.7%)
Student^b^	3 (8.8%)
In retirement^b^	9 (26.5%)
Unemployed^b^	7 (20.6%)
Other^b^	1 (2.9%)
IPAQ-SF ^b^	
Low^b^	15 (44.1%)
Moderate^b^	17 (50.0%)
High^b^	2 (5.9%)
PANSS	
Positive scale^a^	15.9 (5.6)
Negative scale^a^	20.7 (5.6)
SNS ^a^	15.4 (7.3)
Social withdrawal^a^	3.2 (2.2)
Diminished emotional range^a^	3.1 (2.0)
Alogia^a^	3.3 (2.3)
Avolition^a^	3.1 (2.0)
Anhedonia^a^	2.6 (2.0)

*N* = 34, Diagnosis: according to ICD-10; IPAQ-SF: International PA Questionnaire – Short Form; PANSS: Positive and Negative Syndrome Scale; SNS: Self-Evaluation of Negative Symptoms Scale

^a^*M* (*SD*)

^b^*n* (%) if not otherwise specified

### Within subject changes

Table 3 lists all results, including sample sizes, means, standard deviations, and within-subject changes from T0 to T1 with their associated effect size, for each scale.

**Table 3:** Within subject changes from baseline (T0) to post-intervention (T1)

Variable		T_0_		T_1_		95% CI		
	*n*	M	SD	M	SD	t/z	*p*	d_rm_/*r*
**Oxytocin plasma levels (pg/ml)**	34	**1.8**	**0.8**	**2.0**	**0.9**	-2.56	0.045*	**0.90**
IRI								
Fantasy	34	11.7	3.2	11.6	3.0	0.45	0.713	0.10
Empathic concern	34	13.6	2.5	13.6	2.9	0.11	0.911	0.04
Perspective taking	34	13.7	2.7	13.0	2.5	1.59	0.195	-0.33
Personal distress	34	12.0	2.9	11.2	3.2	2.12	0.082	-0.50
Total	34	39.0	7.1	38.2	7.0	1.05	0.426	-0.29
MET								
Cognitive empathy	31	27.7	4.7	27.3	6.4	0.66	0.613	-0.19
**RT**	31	**9148.4**	**4553.7**	**6853.7**	**3503.8**	4.05	0.004**	**-0.98**
Positive emotions	31	14.2	2.6	13.8	3.3	0.94	0.451	-0.28
**RT**	31	**8569.3**	**4469.6**	**6850.5**	**3810.5**	2.69	0.039*	**-0.57**
Negative emotions	30	13.6	2.8	13.9	3.0	-0.63	0.614	0.17
**RT**	30	**9859.6**	**5193.5**	**6741.4**	**3600.6**	5.01	0.000**	**-1.31**
Emotional empathy								
Positive emotions	31	5.4	1.6	5.5	1.4	-0.30	0.797	0.06
**RT**	31	3968.5	2535.9	3309.8	1993.9	2.00	0.101	-0.46
Negative emotions	31	4.8	1.8	4.7	1.9	0.96	0.451	-0.34
**RT**	31	**4986.8**	**3035.3**	**3652.0**	**2274.3**	2.91	0.028*	**-0.56**
PANAS								
Positive affect	30	2.7	0.8	3.0	0.9	-1.76	0.154	0.37
**Negative affect**	30	**1.8**	**0.7**	**1.4**	**0.5**	3.28	0.018*	**-0.53**
Stress and contentment								
General stress^a^	26	2.3	1.6	1.7	1.5	-2.35	0.051	− 0.46
**Symptom-related distress**^**a**^	26	**2.1**	**1.9**	**1.5**	**1.4**	-2.72	0.028*	**− 0.53**
Body wellbeing^a^	22	3.4	1.1	4.0	1.0	-2.21	0.065	− 0.47
**Contentment**^**a**^	22	**3.1**	**1.2**	**4.5**	**1.0**	-3.19	0.008**	**− 0.83**
BMQ								
Experience	33	21.5	7.5	23.2	6.0	1.41	0.251	0.24
Appreciation	33	27.7	8.0	29.9	7.3	-2.13	0.082	0.47

IRI: Interpersonal Reactivity Index; MET: Multifaceted Empathy Test; RT=response time; PANAS: Positive and Negative Affect Scale; Stress an contentment: analog within-session effects; BMQ: Body Mindfulness Questionnaire. *d*_*rm*_ = adjusted Cohen’s *d* for repeated measures [65]. *p* = one-tailed, adjusted for false discovery rate [66], t-test was conducted, if not otherwise specified. ^a^Wilcoxon test was conducted. * *p* < .05, ** *p* < .01, *** *p* < .001

### Oxytocin plasma levels

**Fig. 2:**
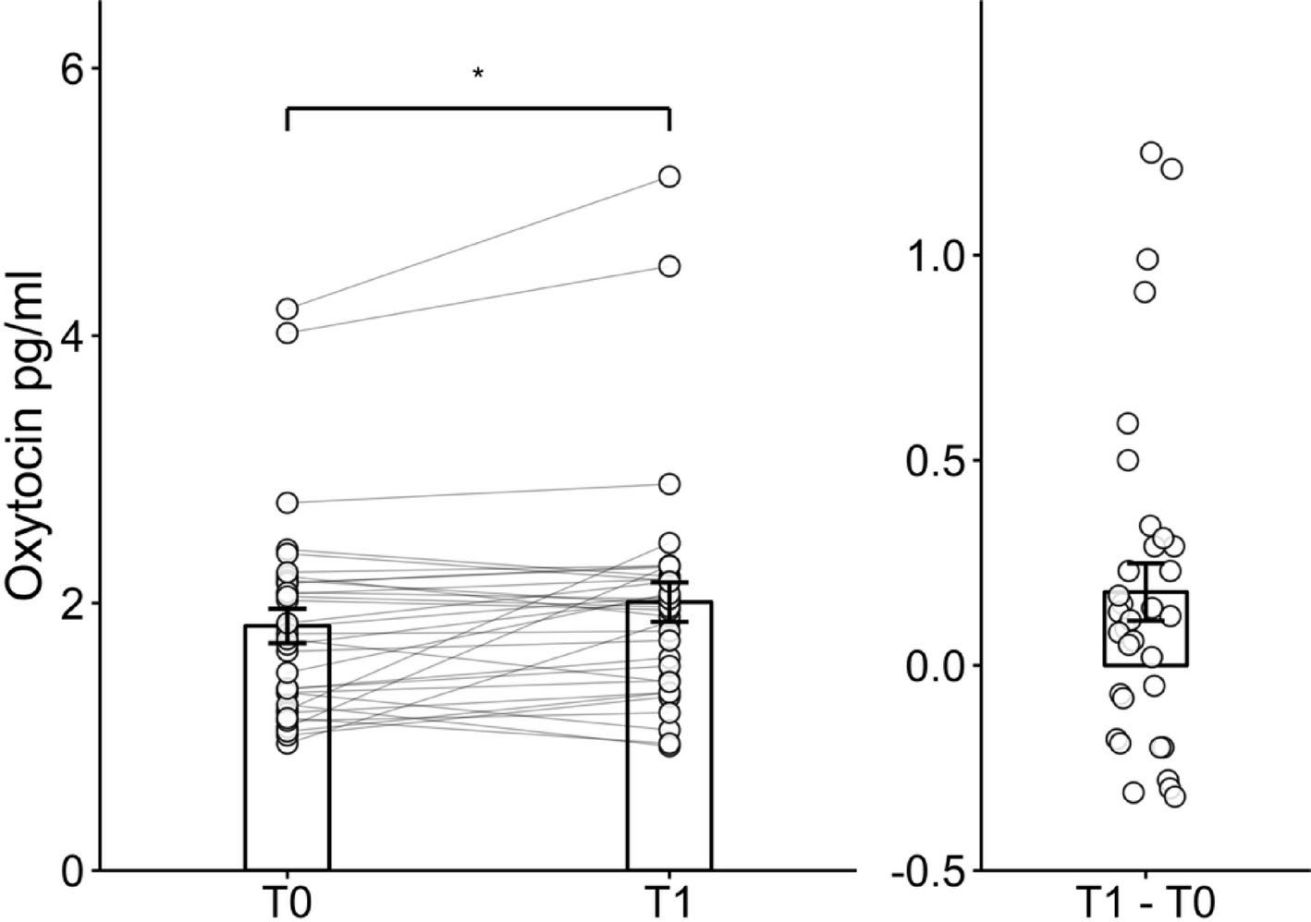
Change scores of oxytocin plasma levels. *Note*: Left panel: paired individual trajectories (grey lines) and individual data points (dots) illustrate within-subject changes from T0 to T1. Right panel: distribution of individual change scores (T1–T0). Bars represent mean ± SEM. *: *p* < .05 (paired-samples t- test)

The oxytocin plasma levels increased significantly by group PA from T0 to T1, *t* (33) = -2.56, *p <* .05, *d* = 0.9, corresponding to a large effect size (see Fig. 2).

### Empathy

Concerning empathy in the IRI, a trend toward a reduction in the *Personal distress* subscale was found with a medium effect, *t*(33) = 2.12, *p* = .082, *d* = -0.50. No changes in other subscales or the total score were observed. In the MET, no changes in cognitive or emotional empathy scales were found, but there were significant reductions in response times from T0 to T1. Since there were no significant empathy changes from baseline to post-intervention, no correlation was calculated between the difference values of empathy and the difference values of oxytocin plasma levels.

### Affect

Regarding affect measured with the PANAS, a significant reduction in *Negative affect* after group PA was reported with a medium effect size, *t*(29) = 3.28, *p* < .05, *d* = -0.53. No improvement in *Positive affect* was observed.

### Stress, well-being and body awareness

Wilcoxon signed-rank tests were performed for changes in stress and contentment, measured with visual analog scales. Outcomes showed significant reductions in *symptom-related distress*, *z* = -2.72, *p* < .05, *r* = -.53, corresponding to a large effect, and a trend toward reductions in *general stress*, *z* = -2.35, *p* = .051, *r* = − .46, with a medium effect. A significant elevation in contentment was observed, *z* = -3.19, *p* < .01, *r* = -.83, implying a large effect, as well as a trend toward enhanced *body well-being z* = 2.21, *p* = .069, *r* = -.47, indicating a medium effect. There was a trend for an increase in *Appreciation of body awareness* measured by the BMQ, *t*(32) = -2.13, *p* = .082, *d* = 0.47 with a medium effect.

### Subjective side effects

Five participants mentioned subjective side effects in the self-rating after the group PA. Four of the participants described these as mild, with a focus on a feeling of exhaustion. For some of these participants, the perception of not being physically fit led to unpleasant thoughts and emotions. One participant reported negative thoughts due to overweight and pain in the joints during exercise. Another participant indicated a somatic side effect of feeling dizzy after 20 min of exercise as a moderate somatic side effect. An overview of the subjective side effects can be found in Table 4.

**Table 4:** Subjective side effect profiles via self-rating by the participants

	*n* (%)	Mild	Moderate	Severe
Subjective side effects	5 (19.2%)	-	-	-
Cognitive	4 (15.4%)	4 (15.4%)	-	-
Emotional	1 (3.8%)	1 (3.8%)	-	-
Somatic	5 (19.2%)	3 (11.5%)	2 (7.2%)	-

*N* = 26 (number of subjects who provided feedback via the questionnaire)

## Discussion

### Physical activity, oxytocin, empathy, stress and their possible associations

Our study found that a brief group-based physical activity (PA) session led to increased oxytocin plasma levels in individuals with SSD, consistent with previous findings showing oxytocin elevations following individual PA [64]. Although most studies have investigated PA in individual formats [37], growing evidence suggests that group PA may yield additional mental health benefits in SSD [41]. While underlying mechanisms remain to be clarified, our results suggest that even low-intensity group PA can enhance endogenous oxytocin levels [47].

These findings are relevant in light of oxytocin’s role as a social-affiliative hormone [16]. Mindfulness- and yoga-based interventions conducted in group settings have similarly shown oxytocin modulation [45, 46]. According to the social-salience hypothesis, oxytocin increases sensitivity to socially meaningful cues; thus, a positive group environment may amplify oxytocin release [31]. This provides a plausible explanation for why group PA may be particularly suited to targeting endogenous oxytocin in SSD, especially regarding its potential links to social-cognitive outcomes such as empathy.

Group PA may influence oxytocin not only through social mechanisms but also through embodied processes. PA targeting body tension and posture can enhance bodily awareness, support emotional resonance, and thereby facilitate empathic processes [39]. Our low-intensity, socially embedded intervention was designed to be feasible for individuals with SSD, who frequently experience motivational difficulties [43], and benefit from structured interpersonal engagement that counteracts social withdrawal and blunted affect [31]. This conceptually grounded and feasibility-oriented intervention will be further evaluated in subsequent studies.

Although negative symptoms were assessed only at baseline and no immediate changes were expected after a single session, oxytocin increases may represent an early neuroendocrine shift relevant for stress buffering and social-affiliative functioning [20, 30, 67]. Given that stress exacerbates negative symptoms, repeated group PA sessions may, over time, link oxytocin-related stress reduction to improvements in empathy and broader psychosocial functioning [37]. Interpretation of effects must remain cautious due to the absence of a control group, as factors such as group cohesion, peer support, and instructor encouragement may also contribute to oxytocin release [68]. Future studies with larger samples, control conditions, and repeated sessions are needed to examine whether changes in oxytocin correlate with improvements in empathy, stress, and negative symptoms, which were not further investigated due to the lack of a significant increase in empathy. A trend toward reduced *Personal distress* on the IRI emerged after the PA session. *Personal distress* is associated with emotion regulation rather than empathic accuracy [56, 69], and reductions are consistent with research indicating that PA can enhance emotion regulation [70], and that oxytocin buffers stress and supports social-affiliative behavior [29, 68].

Together with reductions in *symptom-related distress* and a trend toward lower general stress, these findings suggest that oxytocin-related stress buffering may represent an early effect, whereas improvements in empathy may require repeated sessions or longer training periods, as shown in multi-week PA interventions [37].

No changes were observed in MET cognitive or emotional empathy scores. Shortened reaction times likely reflect task familiarization rather than empathy improvement [37, 38]. While in previous studies shortened reaction times were interpreted as improved empathy [71], the present study lacked control for learning effects, making an empathy improvement interpretation inconclusive. Previous work indicates that group PA can improve empathy only after extended interventions [72], supporting the interpretation that biological shifts (oxytocin) may precede measurable improvements in behavioral empathy.

Interpretation should be cautious due to the single-arm design, as factors such as group cohesion, peer encouragement, and instructor support may have contributed to the oxytocin increase. However, these psychosocial elements may represent integral components of the mechanism rather than confounding variables, given their potential to modulate oxytocin release [68]. PA in a group setting combines embodied activation, social interaction, and motivational reinforcement—elements particularly valuable for individuals with SSD, who often exhibit low motivation for individual PA [43].

Stress is a critical component in SSD and is strongly tied to negative symptoms, affective flattening, and reduced motivation. The observed reductions in *symptom-related distress*, *general stress*, and *Negative affect* further underscore the potential role of oxytocin in short-term stress modulation. Given that stress exacerbates negative symptoms and disrupts social cognition, oxytocin increases during PA may provide a neuroendocrine pathway linking physical activity to improvements in social and emotional functioning [27, 73–75].

We also observed improvements in body awareness, contentment, and body well-being. Although not primary outcomes, these changes may indicate early experiential benefits of PA, which are especially important given the high prevalence of low self-esteem and self-stigma in SSD [76]. Reduced *Negative affect* without increased Positive affect in our exploratory analyses mirrors findings from previous PA studies in SSD and may reflect initial exertion among participants with low baseline PA (44.1% low activity) [77].

Overall, our results suggest that group PA may influence oxytocin, stress, and early emotion-regulation processes, providing a potential foundation for later improvements in empathy and social functioning. Future studies should incorporate control groups, larger samples, and repeated sessions to examine whether changes in oxytocin mediate long-term improvements in empathy, stress, and negative symptoms in SSD.

### Subjective side effects

A few participants self-reported subjective side effects, most of which were somatic and could be attributed to increased exertion durin PA and related to a lack of physical fitness. However, such transient side effects are common and also occur frequently in healthy people during PA [78]. A lower fitness level of some participants should be further considered when adapting the exercises to guide the session. This shows the importance of incorporating PA into the daily lives of individuals with SSD. In addition, the low level of PA emphasizes the importance of reinforcement by positive feedback during the intervention, motivating participants to recognize their efforts. All reported side effects were acceptable and not inconsistent with the implementation of the intervention. All participants remained in the study, indicating high feasibility and acceptability of the intervention by individuals with SSD.

### Limitations

The study used a pre-post-intervention design without a control group, limiting the interpretation of the results. A randomized controlled trial with healthy individuals and treatment as usual control conditions should be conducted to confirm the observed effects and decipher the process mechanisms. Due to the study’s exploratory nature, various constructs were assessed, and multiple tests were conducted with inflated effects being expected [79]. It was also refrained from controlling for characteristics such as age and sex due to the small sample size in the context of a single-arm exploratory pilot study, which should be supplemented in a larger randomised controlled trial.

Therefore, the results should be interpreted with caution and need to be confirmed in future studies. The assessment was relatively long, at 150 min, compared to the 30-minute intervention, and may have attenuated the positive effects of the intervention. Regarding our results, the MET may not be designed to be administered twice within this short period of time. Most instruments have been used in populations with SSD, but cognitive deficits, as a core symptom of SSD, may have influenced the understanding of some questionnaires.

## Conclusion

In conclusion, a single session of group-based PA increased endogenous oxytocin levels in individuals with SSD and was accompanied by reductions in stress-related and negative affective states. These findings support the concept that group PA can engage oxytocin-related neuroendocrine pathways relevant to stress regulation and social-affiliative functioning. While empathy did not improve robustly after one session, early changes in emotion regulation in the form of *Personal distress* suggest that oxytocin-mediated effects may precede measurable improvements in social cognition. Given the feasibility of the intervention and its biological and psychological effects, group PA represents a promising adjunctive approach for SSD [80]. Future randomized controlled trials with repeated sessions are needed to determine whether changes in oxytocin mediate longer-term improvements in stress, empathy, and negative symptoms.

## Supplementary Information

Below is the link to the electronic supplementary material.

Supplementary Material 1

## Data Availability

The data supporting this study’s findings are available from the corresponding author upon reasonable request.
