# Supplementary material for: The influence of group physical activity on oxytocin, empathy, affect, and stress in individuals with schizophrenia spectrum disorders in a single-arm, pilot and exploratory study
Source: Discov Ment Health. 2026 Mar 12;6(1):59. doi: 10.1007/s44192-026-00420-9 (PMC13066114; doi:10.1007/s44192-026-00420-9)
Supplement: Supplementary file 1 — Supplementary Material 1 [file 44192_2026_420_MOESM1_ESM.docx]

**Supplementary Material**

**Supplementary Table 1**

*Description and Instructions of Exercises of the Intervention in Order of Execution*

|  | Exercise | Aim | Explanation/ Attention focus |
| --- | --- | --- | --- |
| **Introduction**  3 minutes | Saying the name and reporting on how the patients are feeling at the moment. | - Getting to know each other  - Preparing mentally to exercise  - Creating group feeling |  |
| **Circulation/**  **Activation**  3 minutes | 1. **Running on the spot**, approx. hip width (walking)  2. **Tiptoes running** inwards and outward (5x)  3. **Run into wider stance**, then close legs again (5x) | - Increase circulation  - Focus attention on the body  - Mobilize Feet / ankle joints (inversion /eversion / dorsiflexion /plantar flexion) and activate musculature | Thighs roll in the hip inwards and outwards, we knead the feet and prepare them for the workout.  Point out heat, circulation, heart  Methodical series:  1. Tiptoe inward turn ("snowplow", "urgent need to go to the toilet"), then ball run ("tiptoeing")  2. Turn toes outwards (“duck walk”), then ball run  3. Alternating internal/external rotation |
| **Mobilization**  5 minutes | 1. **Alternation toe stand/heel stand** (8-10x)  2. **Upper body rotation** around longitudinal axis (5x per side)  3. **Lateral flexion** (upper body lateral tilt - 3x per side, hold 5 sec each)  4. **Flexion /extension** (3x) (torso flexion forward / max. extension) | - Mobilization of the spine in all levels of movement  - Activation of the  trunk muscles  - "Sensing" the musculature | 1. We go into the toe stand, take the arms stretched above the head and lean backwards / push the pelvis forward ("As tall as possible") Knees are always stretched. Then roll back onto heels and lower arms  2. Upper body as long as possible, gaze remains directed forward, arms swing loosely over shoulders to the back  3. Stand hip-width apart, right arm stretched close to the head. Weight on right leg (push pelvis to right side), arm over head to the left out of the shoulder – stretch from shoulder over side to pelvis noticeable? With exhalation back to the center, than to the other side  4. Bend the upper body with bend knees forward and down. 3x inhale and exhale deeply, reaching to the floor with exhale. Then straighten up, arms overhead, hold 2 sec in extension |
| **Coordination/**  **Balance**  4 minutes | **One leg stand** (right / left)  Three levels:  1. Stand on one leg, toes of other foot are on the floor, and carries little weight  2. Second foot off the floor  3. One-leg stand: slowly alternate gaze to the floor and to the ceiling (difficult) | - Improvement of balance ability  - Conscious control of the trunk muscles | Slight bending in the knee of the active leg, upright posture (pull shoulders back and down, tense abdomen) With the foot of the active leg grasp into the floor/sole of the shoe. ("Hold on like a little bird on branch")  Alternating sides, each side hold approx. 20 sec.  First round: toes of the lifted leg still touch the ground to stabilize the posture  Second round: foot is fully lifted  Third round: looking to the ceiling / floor bring head back to neutral position and hold briefly |
| **Strengthening**  10 minutes | 4 Exercises:  1. **Squat**, hands grasp at bend forward-upward 15 reps. (go down sufficiently)  *Followed by*: 10x arm circles forward  2**. Lunge** with arms vertically above head, 10x per leg  *Followed by*: 10 x arm circles backwards (big)  3**. Strengthening shoulder girdle/neck**, 90° abduction: 10x Retroversion + Elevation with external rotation  4. **Static squat** (slightly springy) Followed by: Toe stand/fheel stand 5 x alternating | 1. Dynamic training especially for quadriceps femoris and gluteal muscles with extension posture of the spine (strengthening of e.g. erector spinae)  2. Dynamic training especially for quadriceps femoris and gluteal muscles with extension posture of the spine  3. Strengthening rotator cuff, deltoideus, trapezius (combination isometric and dynamic muscle work)  4. Isometric training legs | 1. Shoulder wide stance, pelvis in downward movement back, weight mainly on heels.  Picture: "far behind you is a low chair, short above chair, which you are trying to reach", "skier in downhill crouch".  2. Feet parallel, put one foot back ("to the knight's throne",  drop knee to the floor, over stand up again over the front leg (if possible without help from the back leg)  3. Arms are stretched out to the sides, palms face front, back of the hand runs a quarter circle, back up so that palms face ceiling, shoulder blades pull towards each other. Shoulders remain low, look forward.  4. Picture: skier in downhill crouch, ski jumper on bench before the jump |
| **Warm down**  3 minutes | **Mobilization of the cervical spine** in all planes of motion  (rotation / lateral flexion / flexion and extension) without including shoulders  Rotation, lateral flexion and holding for 20-30 sec each. Only short look to ceiling (2-3 sec)  **Roll shoulders** | - Come to rest  - Increase mobility of the cervical spine  - Counteract tension in the neck | "Look as far back as possible over the shoulder" / "Ear towards the shoulder", "The other ear wants to get as far as possible away from the shoulder".  Chin to chest - look to the ceiling (head to the neck) |
| **Feedback**  2 minutes | Shortly reporting on how you are feeling at the moment | - Feel inside your body  - Closing session |  |
| ***General recommendations***  - All exercises should be done very slowly.  - Consideration should be given to any physical limitations of the patient.  - Biofeedback should be given during exercising (*Describe what you feel*).  - Instructor participates as a movement model (pre-gymnast). Correct your own movements as an instruction to the participants. Describe what you are doing while you act like a movement model.  - Always point out regular breathing: Exhale during tension, inhale when releasing.  - Always point out posture and body tension (shoulders back-down, spine long, abdomen tense).  - For each exercise, name the main muscle group that is being strengthened and point out the purpose of the exercise, e.g. leg training is important, because it is the basis for safe and powerful walking and running.  - Positive reinforcement during the exercises:  - Closing the session with feedback | | | |
